# Retraction: FKBP14 overexpression contributes to osteosarcoma carcinogenesis and indicates poor survival outcome

**DOI:** 10.18632/oncotarget.28697

**Published:** 2025-02-28

**Authors:** Zhongming Huang, Junhua Li, Shaohua Du, Yanghua Tang, Ligang Huang, Luwei Xiao, Peijian Tong

**Affiliations:** ^1^Department of Orthopaedic Surgery, Affiliated Jiangnan Hospital of Zhejiang Chinese Medical University, Hangzhou 310053, China; ^2^Department of Orthopaedic Surgery, Xiaoshan Chinese Medical Hospital of Zhejiang Chinese Medical University, Hangzhou 310006, China; ^3^Zhejiang Chinese Medical University, Hangzhou 310053, China; ^4^Institute of Orthopaedics and Traumatology of Zhejiang Province, Hangzhou 310053, China; ^5^Department of Orthopedic Surgery, The Second Affiliated Hospital, School of Medicine, Zhejiang University, Hangzhou 310053, China; ^6^Department of Orthopaedic Surgery, The First Affiliated Hospital of Zhejiang Chinese Medical University, Hangzhou 310053, China

**This article has been retracted:** Oncotarget has concluded its investigation of this paper. The following was discovered: In Figure 7C, the MMP2 blot image is a duplicate of one found in Figure 6A of another paper published concurrently [1]. The flow cytometry image of UO293 cells NC in Figure 5A is also found in Figure 3D of an earlier published paper [2]. Additionally, some western blot images have been reproduced in a few later published unrelated articles. The corresponding author Peijian Tong has acknowledged that they identified inconsistencies in the data that they have been unable to verify or replicate through further experimentation. Given these findings, the Editorial Board has decided to retract the paper. All authors have agreed with this decision.

Original article: Oncotarget. 2016; 7:39872–39884. 39872-39884. https://doi.org/10.18632/oncotarget.9524
